# Supplementary material for: Inflammatory factors and risk of lung adenocarcinoma: a Mendelian randomization study mediated by blood metabolites
Source: Front Endocrinol (Lausanne). 2024 Aug 27;15:1446863. doi: 10.3389/fendo.2024.1446863 (PMC11384989; doi:10.3389/fendo.2024.1446863)
Supplement: Supplementary file 1 [file DataSheet1.zip › supplementary files/S3/GCST90274801_GCST90199955/sensitivity-analysis.pdf]

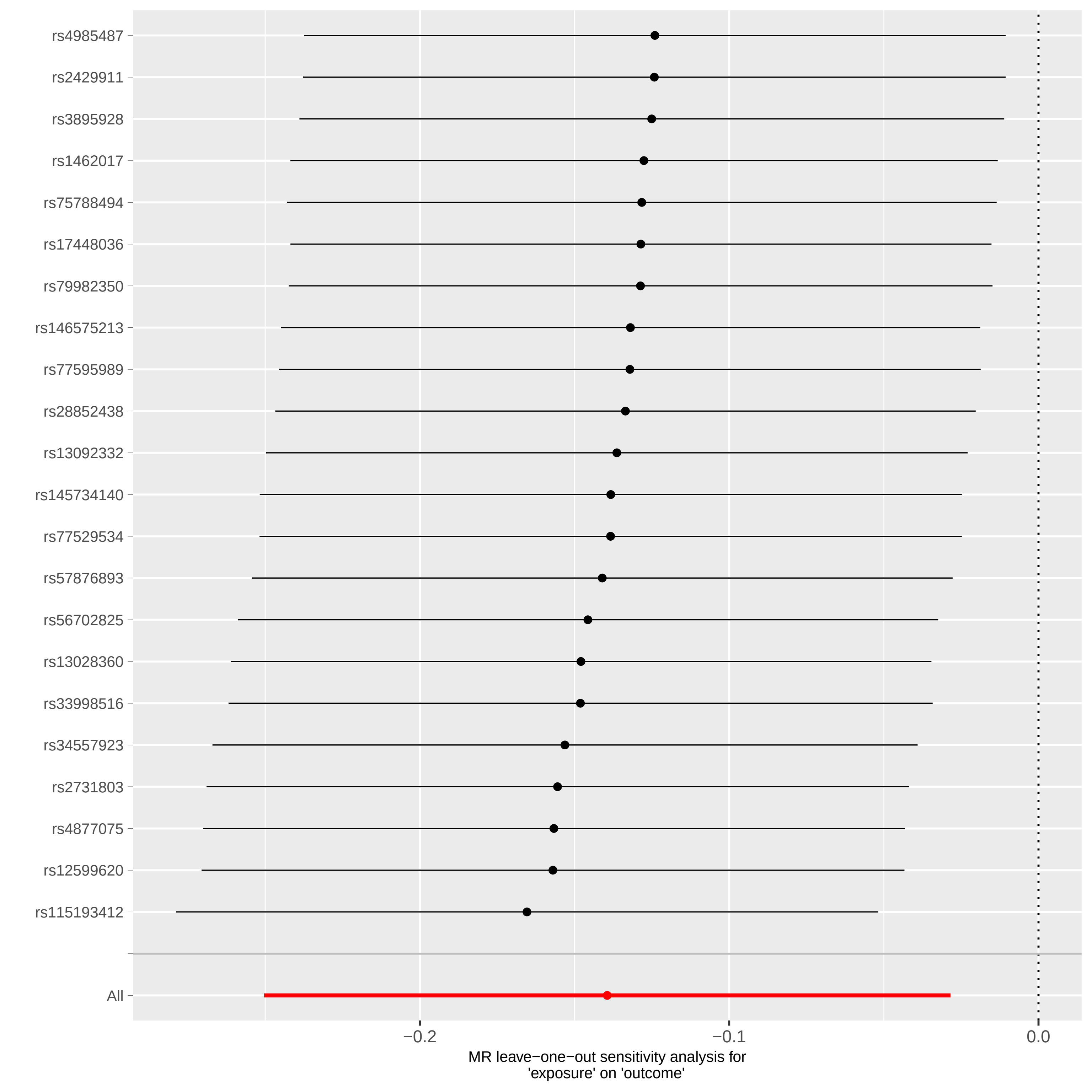
rs4985487
rs2429911
rs3895928
rs1462017
rs75788494
rs17448036
rs79982350
rs146575213
rs77595989
rs28852438
rs13092332
rs145734140
rs77529534
rs57876893
rs56702825
rs13028360
rs33998516
rs34557923
rs2731803
rs4877075
rs12599620
rs115193412
All
−0.2
−0.1
0.0
MR leave−one−out sensitivity analysis for
'exposure' on 'outcome'
